# Simultaneous Administration of ADSCs-Based Therapy and Gene Therapy Using Ad-huPA Reduces Experimental Liver Fibrosis

**DOI:** 10.1371/journal.pone.0166849

**Published:** 2016-12-16

**Authors:** Alejandra Meza-Ríos, Leonel García-Benavides, Jesus García-Bañuelos, Adriana Salazar-Montes, Juan Armendáriz-Borunda, Ana Sandoval-Rodríguez

**Affiliations:** 1 Institute for Molecular Biology in Medicine, Department of Molecular Biology and Genomics, Health Sciences University Center, University of Guadalajara, Guadalajara, Jalisco, Mexico; 2 Unit of Cardiovascular Investigation, Department of Physiology, Health Sciences University Center, University of Guadalajara, Guadalajara, Jalisco, Mexico; 3 INNOVARE, Guadalajara, Jalisco, Mexico; University of Navarra School of Medicine and Center for Applied Medical Research (CIMA), SPAIN

## Abstract

**Background and Aims:**

hADSCs transplantation in cirrhosis models improves liver function and reduces fibrosis. In addition, Ad-huPA gene therapy diminished fibrosis and increased hepatocyte regeneration. In this study, we evaluate the combination of these therapies in an advanced liver fibrosis experimental model.

**Methods:**

hADSCs were expanded and characterized before transplantation. Ad-huPA was simultaneously administrated via the ileac vein. Animals were immunosuppressed by CsA 24 h before treatment and until sacrifice at 10 days post-treatment. huPA liver expression and hADSCs biodistribution were evaluated, as well as the percentage of fibrotic tissue, hepatic mRNA levels of Col-αI, TGF-β1, CTGF, α-SMA, PAI-I, MMP2 and serum levels of ALT, AST and albumin.

**Results:**

hADSCs homed mainly in liver, whereas huPA expression was similar in Ad-huPA and hADSCs/Ad-huPA groups. hADSCs, Ad-huPA and hADSCs/Ad-huPA treatment improves albumin levels, reduces liver fibrosis and diminishes Collagen α1, CTGF and α-SMA mRNA liver levels. ALT and AST serum levels showed a significant decrease exclusively in the hADSCs group.

**Conclusions:**

These results showed that combinatorial effect of cell and gene-therapy does not improve the antifibrogenic effects of individual treatments, whereas hADSCs transplantation seems to reduce liver fibrosis in a greater proportion.

## 1. Introduction

Advanced liver fibrosis (ALF) is a major health worldwide problem and liver transplantation is the ultimate treatment for ALF, although it is infrequent due to low availability of organs, high cost and the risk of transplant rejection. Alternative therapies for liver fibrosis have been developed including stem cell therapy and gene therapy. Mesenchymal stem cells (MSCs) reside in various tissues, such as bone marrow, umbilical cord blood, placenta, liver, adipose tissue and others. MSCs undergo self-renewal but can differentiate into multiple cell lineages; and have demonstrated immunomodulation, inflammation suppression and antifibrogenic effects [1–5]. MSCs isolated from adipose tissue are called adipose derived stromal cells (ADSCs). MSCs, particularly ADSCs, are capable of differentiating into several tissue lineages, including hepatocyte-like cells [6–8]. However, it is unlikely that MSC-derived hepatocyte-like cells could provide sufficient replacement of lost liver function in any given clinical scenario. It has been postulated that cytokine production may be the major mode of therapeutic action of MSCs in advanced liver fibrosis. ADSCs secretome include multiple growth factors, such as VEGF, HGF, GM-CSF, bFGF, BDNF and IGF-1 and interleukins, such as IL-6, IL-7, IL-8, IL-11, IL-10 [8,9]; some of which are suitable inducers of liver regeneration. ADSCs-derived IL-10 and TNFα inhibit the proliferation of hepatic stellate cells and MSC-derived HGF was shown to induce the apoptosis of hepatic stellate cells in a co-culture system [10].

On the other hand, our research group has demonstrated that urokinase Plasminogen Activator (uPA)-gene therapy is powerful resource for activating latent metalloproteinases and single chain-HGF, promoting extracellular matrix degradation and hepatic regeneration [11,12]. Ad-huPA administration reduced fibrotic tissue by 50–60% and induced brisk hepatocyte regeneration in CCl_4_-induced cirrhosis [12]. Adenoviruses have been shown to be efficient vectors in cirrhosis models, introducing exogenous cDNA into the liver [13]. The aim of this study was to evaluate the combination of cell therapy with ADSCs and gene therapy using Ad-uPA in the resolution of fibrosis and comparing the results with the effects of individual therapies in an experimental model of liver fibrosis.

## 2. Materials and Methods

### 2.1 hADSCs Isolation and culture

Human adipose tissue was obtained from abdominal fat of females subjected to cosmetic liposuction who signed a written consent to donate the discarded fat tissue. The protocol was approved by the Research and Ethical Committee of the CUCS, Universidad de Guadalajara (approval number C.I. 067–2012) which review the fat obtainment procedure. Tissue was digested by a 0.075% collagenase type II solution (Invitrogen, Grand Island, NY) for 1 hour at 37°C with gentle shaking. Digestion product was filtered using a 100 um nylon mesh and centrifuged at 1200 g for 8 min. Pellet was washed with PBS once. Cells were collected and plated on plastic dishes in DMEM (Invitrogen, Grand Island, NY) supplemented with 10% fetal bovine serum (Invitrogen, Grand Island, NY) and 1% antibiotic (Invitrogen, Grand Island, NY). The medium was changed after 48 h. Cells were harvested and seeded until passage three for achieving greater expansion. Cell characterization and transplantation was made using a unique batch of cells from a sole isolation.

### 2.2 ADSCs characterization and *in vitro* differentiation

Surface markers for ADSCs were evaluated using a mini Guava EasyCyte flow cytometer. 5x10^4^ cells were incubated with fluorescein isothiocyanate (FITC)- or phycoerythrin (PE)-conjugated antibodies, anti-CD105, anti-CD34, anti-STRO, anti-CD73, anti-CD45, anti-HLA-ABC, anti-HLA-DR (Invitrogen, Frederick, MD) for 30 min at 4°C in PBS and washed afterwards. Cell autofluorescence in channel F1 or F2 was subtracted for obtaining the neat signal of each marker. 5x10^4^ cells were plated for differentiation tests. Adipogenic differentiation was performed in StemPro adipogenesis conditioned media (Invitrogen, Grand Island, NY) for one week. For validating differentiation, lipid droplets were stained using Oil red staining. StemPro osteogenesis conditioned media (Invitrogen, Grand Island, NY) was used for osteogenic differentiation for 11 days, replacing media every 48 hours. Extracellular calcium deposits were stained by Von Kossa. Hepatogenic differentiation started with 2 days of pre-culture in DMEM enriched with EGF and bFGF (Invitrogen, Grand Island, NY), followed by 7 days cultured in HGF, bFGF and nicotinamide enriched media. Subsequently, cells were cultured 12 days in media containing OSM (Recombinat human Oncostatin M) (Invitrogen, Grand Island, NY), dexametasone (Bio Vision, Milpitas, CA) and ITS (mixture of recombinant human insulin, human transferrin, and sodium selenite) (Sigma-Aldrich, St. Louis, MO). Media was changed 2 times per week. Albumin and α-fetoprotein expression were determined by anti-albumin (Dako Denmark A/S, Glostrup, Denmark) and anti-α-fetoprotein fluoresce-conjugated antibodies (Thermo Scientific, Rockford, IL).

### 2.3 Ad-huPA production

Ad-huPA is a first generation recombinant human Ad-serotype 5 with huPA cDNA driven by CMV promoter. Ad-huPA was produced by a recombination of pMH4-huPA and pJM17 ΔE1 Ad-v backbone, resulting in a 41.4 kb Ad-vector (Ad-huPA). Ad-huPA was amplified in HEK293 cells -DMEM 10% FBS, 37°C and 5% CO_2_ atmosphere. Cells were collected after 48 h of transduction disrupting cells in three cycles of freeze/thawing for initiating rAd purification. Cellular debris was removed by centrifugation and supernatant containing rAd was concentrated by two ultracentrifugation rounds at 141,000 RCF in a CsCl density gradient. Ad-huPA particles per milliliter were determined by optical densitometry (OD), and infection units using end-point titration [14].

### 2.4 Animal model of advanced liver fibrosis and therapy administration

Male Wistar rats (n = 10/group, ~120 g) were intoxicated i.p. with CCl_4_ for 8 weeks. Non-manipulated rats served as healthy control (n = 10). Since ADSCs come from a xenogenic source, cirrhotic together with healthy animals were immunosuppressed using 10mg/kg/day of Cyclosporine A (orogastrically) until sacrifice. 2x10^6^ hADSCs and/or 3x10^11^ vp of Ad-huPA were administrated via iliac vein 48 h after first CsA administration. Animals were sacrificed 10 days posttreatment; hepatic tissue and blood were collected. A sub-group of cirrhotic rats were transplanted with DAPI-stained hADSCs for determining biodistribution. Animals were obtained from the Animal facility of the Health Sciences University Center of the University of Guadalajara and housed in a maximum of 4 animals/cage. Rats received care according to the Mexican Official Norm NOM-062-ZOO-1999 and guidelines of the Animal facility of the Health Sciences University Center of the University of Guadalajara using 12h light/dark cycles. Rats were fed *ad libitum* and had free access to water. Health of the animals was monitored daily. When animals were identified in pain during CCl_4_ intoxication, had ear infection, showed slow or not movement, had brittle hair and eye dehidratation; animals were euthanized using excess of anesthesia. No animals died prior to the experiment endpoint. The protocol was approved by the Research and Ethical Committees of CUCS, Universidad de Guadalajara (approval number C.I. 067–2012) which reviewed and approved the animal mortality aspects of the protocol.

### 2.5 Analysis of liver tissue specimens

Representative tissue from the five liver lobules was removed after sacrifice and fixed by immersion in 10% paraformaldehyde solution, dehydrated in ethylic alcohol, and embedded in paraffin. Sections 5μm thick were stained with Masson´s trichrome and Hematoxylin & Eosin. Percentage of fibrotic tissue was determined in 30 microphotographs using a computer-assisted image analyzer (Image-ProPlus 6.0, Media Cybernetics, Inc., Bethesda, MD). Immunohistochemistry was performed in liver sections incubated with monoclonal antibody against α-SMA (Dako Denmark A/S, Glostrup, Denmark) diluted 1:100 in PBS. Incubation with a biotinylated secondary antibody (Vector, Burlingame, CA) was revealed with 3,30-diaminobenzidine (Sigma Aldrich, St. Louis, MO). Percentage of α-SMA reactivity was determined in 15 microphotographs using a computer-assisted image analyzer (Image-ProPlus 6.0, Media Cybernetics, Inc., Bethesda, MD). Necroinflammation index and fibrosis staging were measured by a hepato-pathologist blinded to the study using the HAI index [15]. Liver tissue RNA isolation was performed according to the Chomczynski and Sacchi modified method [16]. Briefly, liver tissue was homogenized in the presence of a Trizol reagent (Invitrogen, Carlsbad, CA). Chloroform was added and the aqueous phase was isolated. RNA was precipitated with isopropanol. RNA quantity and quality were determined in NanoDrop equipment (Thermo Scientific, USA). For retrotranscription, 2 μg of total RNA were used with 240 ng Oligo dT, 0.5 mM dNTPs mix, 10mM DTT, 2 U of RNAse inhibitor and 200 U MMLV(Invitrogen, Carlsbad, CA). Incubation was performed for 10 min at 25°C, 50 min at 37°C, 15 min at 70°C and 5 min in ice. The samples were stored at -70°C until used. Using a LightCycler 96 instrument (Roche Diagnostics, Indianapolis, IN) the qPCR was performed as follows: 1 cycle at 50°C for 2 min, 1 cycle at 95°C for 5 min, and 30–40 cycles at 95°C for 30 s and 60°C for 40 s. The total reaction volume was 10 μL containing 2 μL of cDNA, 1X Universal PCR Master Mix (Roche, Branchburg, NJ) and 1X TaqMan primers/probe (Applied Biosystems, Foster City, CA). Levels of mRNA of Col1a2, Tgfb1, Ctgf, Mmp2, Acta2 and PAI1A were normalized using glyceraldehyde phosphate dehydrogenase (Gapdh) as housekeeping gene. See S1 File for catalog number and sequence of TaqMan Probe.

Proteins were extracted from 100–150 mg of liver minced in a buffer solution (pH 7.4) containing 65 mmol/L Tris, 310 mmol/L KCl, 1% Nonidet P40, 0.1% SDS, sodium orthovanadate 200 mmol/L, 1 mol/L NaF and 1X complete protease inhibitor cocktail tablets (Roche Diagnostics, Indianapolis, IN). After centrifugation at 21200 RCF /4°C for 1 hour, supernatant was isolated and stored at -80°C until required. The total protein quantification was performed using a Bradford assay [17]. huPA was quantified using a ELISA (Abcam Plc, Cambridge, UK) assay according to the manufacturer instructions.

### 2.6 Blood sample determinations

Blood was collected when the animals were sacrificed and serum was obtained. Serum levels of alanine aminotransferase (ALT), aspartate aminotransferase (AST) and albumin were measured in an automated Vitros DT 60 equipment (Johnson and Johnson, New Jersey, NY).

### 2.7 Statistical analysis

Values are expressed as median, or as mean ± SEM. Groups were compared with the Kruskal Wallis test. Differences between two groups were analyzed with the Mann Whitney U test. Values of p <0.05 were considered statistically significant. Analysis was run with SPSS 18.0. Detailed data are available in S2 File.

## 3. Results

### 3.1 Characterization of hADSCs expanded in vitro

**Fig 1:**
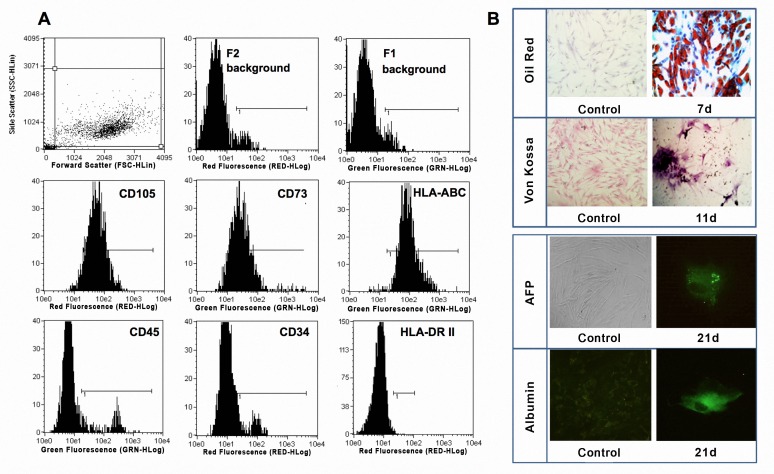
Characterization of hADSCs. **(A)** Phenotypic characterization of ADSCs showed cell markers CD105, CD73 and HLA-ABC positive in the population. CD45, CD34 and HLA-DR resulted negative in selected population. **(B)**Functional characterization of ADSCs into adipogenic, ostegenic and hepatic linage. Photographs (40X) showed non-differentiated cells (control) used as controls of staining and IF and cells positive for lipid droplets (oil red), extracellular calcium deposition (Von Kossa) and IF for AFP and Albumin (FITC).

After expansion to passage 3 in supplemented-DMEN, ADSCs exhibited a characteristic elongated fibroblast-like morphology. Cell surface marker analyses by flow cytometry revealed that CD105, CD73 and HLA-ABC were positive in expression and that CD34, CD45 and HLA-DR were negative in expression “Fig 1A”. The ability of these cells to differentiate into osteoblasts, adipocytes and hepatocyte-like cells was confirmed. Fig 1B represents immunocytochemical evidence of ADSCs differentiation into hepatocyte-like cells showing fluorescence with anti-albumin or anti-AFP. Adipogenic differentiated cells accumulated intracellular lipid droplets stained with oil red, whereas extracellular calcium phosphate deposits were observed in osteoblast differentiated cells using Von Kossa staining.

### 3.2 In vivo tracking of hADSCs in fibrotic rats

**Fig 2:**
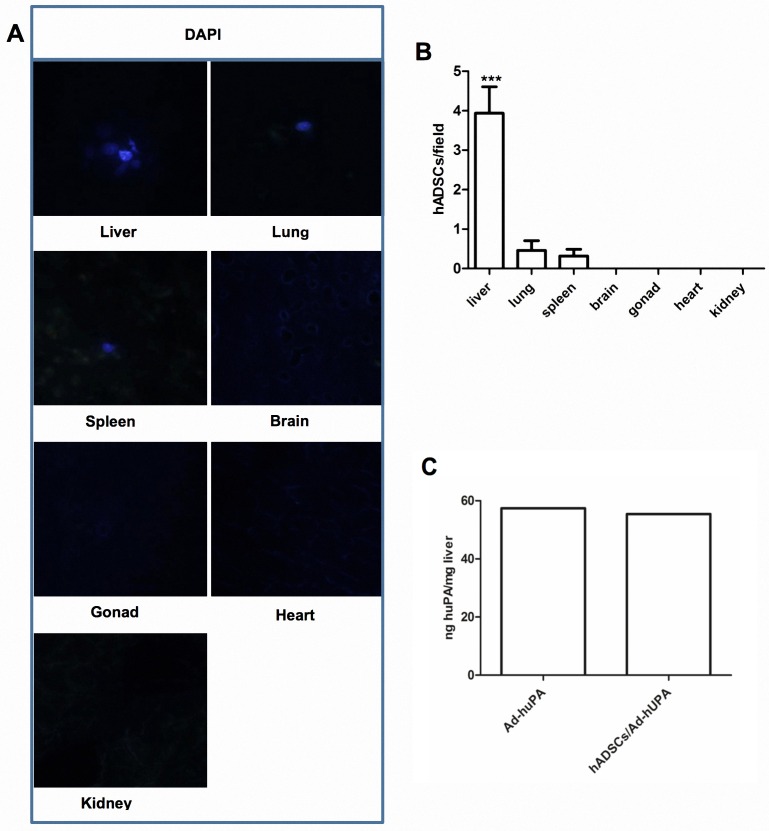
Biodistribution of hADSCs in cirrhotic rats. **(A)** DAPI-stained cells transplanted via iliac vein were detected mainly in liver, and in a very low frequency in lung and spleen. **(B)** Quantification of DAPI-positive cells per field of view in collected organs (***P<0.001). **(C)** huPA protein levels in liver homogenates of cirrhotic rats treated with Ad-huPA or the combination of hADSCs and Ad-huPA. Data represent the median for each group.

To investigate biodistribution of hADSCs in cirrhotic animals after systemic transplantation, rats were sacrificed 10 days after DAPI-stained cell transplantation. Liver, lung, heart, spleen, kidney, brain and testis were analyzed. Detection of the DAPI fluorescence signal revealed that hADSCs migrated to the liver and some of them were arrested in lung and spleen, as seen in Fig 2A and 2B.

### 3.3 Expression of huPA protein in cirrhotic liver of animals treated with Ad-huPA or hADSCs/Ad-huPA

huPA protein was quantified by ELISA assay for confirming the transduction of Ad-huPA into the liver of cirrhotic animals and evaluating possible differences in transgene expression between treatments. Human uPA protein was detected in liver homogenate of animals treated with gene therapy or the combination of gene and cell therapy. The average levels of expression in liver homogenates for Ad-huPA group was 66.44± 18.50 ng/mg and 65.87± 16.69 ng/mg of tissue in the group receiving combined therapies. No statistical difference between groups was achieved, which demonstrates that transgene expression was similar between groups, even when they received either gene therapy alone or combined with hADSCs “Fig 2C”.

### 3.4 Individual therapies and their combination diminished liver fibrosis, improved inflammation and fibrosis score and reduced hepatic expression of α-SMA protein and Sirius red collagen staining

**Fig 3:**
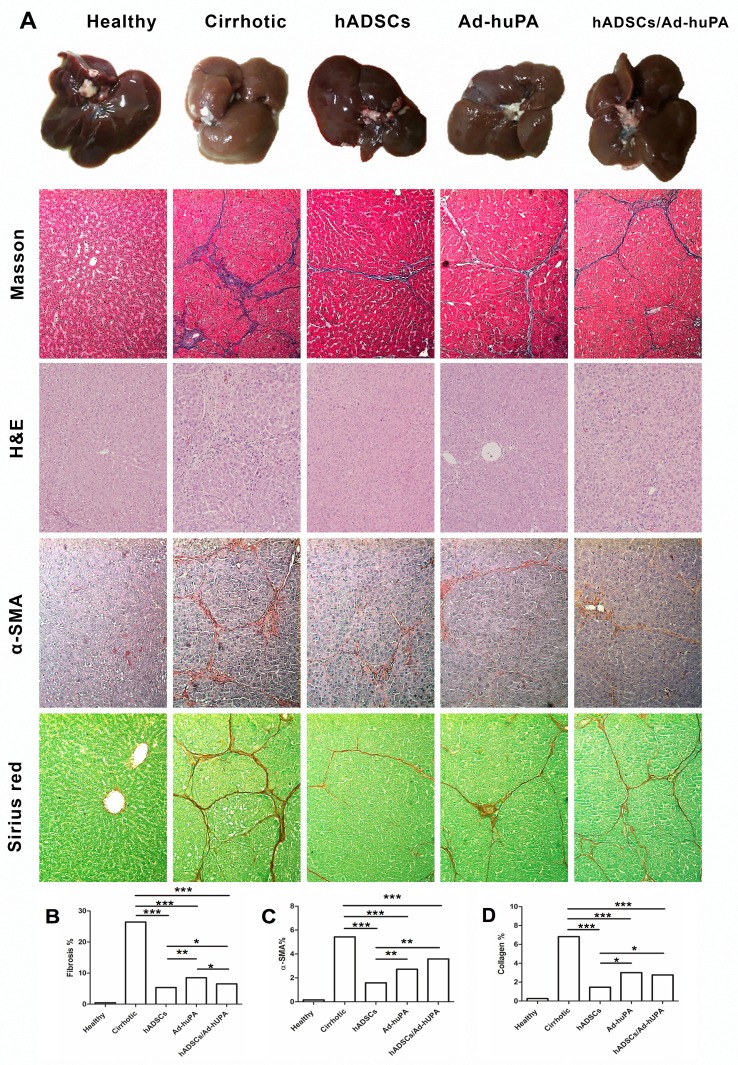
Assessment of liver fibrosis, inflammation, α-SMA and collagen staining in cirrhotic animals treated with cell and gene therapy. **(A)** Macroscopic appearance of the liver and Masson staining showed characteristic morphological liver alterations in cirrhotic rats. Animals treated with any of the therapies showed reduction of fibrotic tissue and decrease in inflammatory cell infiltrate in H&E-stained liver. IHC for **α**-SMA revealed decrease in staining in treated animals. Sirius red staining indicates a reduction in liver collagen content in animals that received treatment. **(B)** Fibrosis percentage in liver tissue was measured in control and treated rats **(C)** Quantification of **α**-SMA immunoreactivity in liver tissue of treated animals and controls. Data represent the median for each group (*P<0.05, **P<0.01, ***P<0.001). **(D)** Morphometric quantification of collagen staining using Sirius Red in liver tissue. Data represent the median for each group (*P<0.05, **P<0.01, ***P<0.001).

Therapies were systemically infused into cirrhotic animals for addressing the anti-fibrogenic effect of hADSCs, Ad-huPA or their combination. The percentage of fibrotic hepatic tissue was calculated in Masson stained liver sections using a computer-assisted morphometric analysis. In cirrhotic controls, characteristic fibrotic septa forming bridges between portal tracts (HAI score 5.57±0.20) and central veins were observed. In treated animals, fibrosis regression was induced, compared to cirrhotic controls as observed in Fig 3A. When examined by a pathologist blind to the study, the livers of treated animals were determined to have reduced bridging fibrosis (HAI score 2.60±0.22 hADSCs, 2.75±0.25 Ad-huPA and 2.62±0.26 hADSCs/Ad-huPA). Slight but statistical significant differences were found between treatments. hADSCs therapy induced fibrosis regression by 78.9±1.9%, Ad-huPA treatment induced a 65.2±2.1% fibrotic tissue regression. Interestingly enough, the combination of cell and gene therapy achieved a 72.0±1.4% fibrotic tissue regression. “Fig 3B”. This piece of evidence deserves further consideration.

Liver samples were stained with H&E for evaluating histopathologic changes “Fig 3A”. H&E staining of liver tissue from cirrhotic controls demonstrated cellular damage and centrilobular necrosis with intense neutrophilic infiltration of inflammatory cells [Histopathological Activity Index (HAI) 7.71 ± 0.28]. Necroinflammation index was improved by all therapies as shown in Table 1. HAI score was reduced by Ad-huPA to 6.62 ± 0.26, by ADSC transplantation to 5.60 ± 0.22 and by the combination of therapies to 5.87 ± 0.29.

**Table 1:** Necroinflammation index and fibrosis staging.

Group	Necroinflammation (0–18)	Fibrosis (0–6)
**Healthy**	0.37 ± 0.18	0
**Cirrhotic**	7.71 ± 0.28	5.57 ± 0.20
**hADSCs**	5.60 ± 0.22 ^a^^***,^ ^b^^**^	2.60 ± 0.22 ^a^^***^
**Ad-huPA**	6.62 ± 0.26 ^a^^*^	2.75 ± 0.25 ^a^^***^
**hADSCs/Ad-huPA**	5.87 ± 0.29 ^a^^**^	2.62 ± 0.26 ^a^^***^

^a^ Statistical significance compared to cirrhotic controls

^b^ statistical significance compared to the Ad-huPA group. Data represent the mean ±SEM for each group

(*P<0.05, **P<0.01, ***P<0.001).

**Fig 4:**
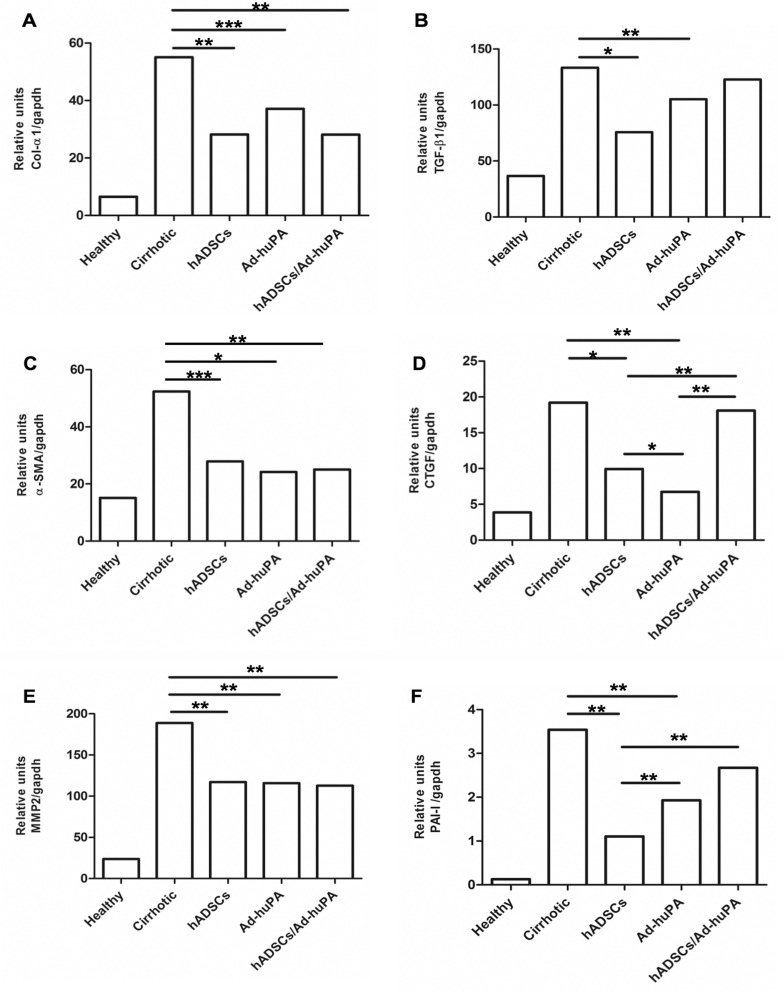
Analysis of profibrogenic gene expression. Mean relative expression levels of **α**-SMA, Col-**α**1, CTGF, MMP2, PAI-I and TGF-β1 in liver tissue. Data are normalized to GAPDH expression levels and represent the median for each group (*P<0.05, **P<0.01, ***P<0.001).

Immunohistochemistry assays were performed to evaluate α-SMA protein expression in liver sections. α-SMA detection reveled an intense staining within the fibrotic septum in cirrhotic controls. Administration of Ad-huPA, hADSCs and hADSCs/Ad-huPA decreased α-SMA staining as shown in microphotographs “Fig 3A”. α-SMA stained area was quantified using Image-ProPlus 6.0 software. hADSCs transplantation reduced α-SMA staining 3.7 fold (P>0.001), Ad-huPA treatment 2.2 fold (P>0.001) and hADSCs/Ad-huPA 1.6 fold (P>0.001), compared to cirrhotic controls “Fig 3A”. This reduction in α-SMA staining correlates with a decrease in α-SMA mRNA levels detected in liver homogenates “Fig 4C”, and it can be speculated that this reduction may be related to a diminution in the quantity of activated HSC, a molecular mechanism that has been described for hADSCs and Ad-huPA treatments.

Sirius red staining of the liver was performed to assess collagen distribution “Fig 3A”. Cirrhotic controls revealed extensive collagen bundles deposition and portal-central and central-central bridges were evident, resulting in a distorted lobular architecture. Morphometric quantification revealed a significant reduction (P>0.001) of the 1.55±0.50% stained area in the hADSCs treated animals, 2.77±0.82% in Ad-huPA animals and 2.45±0.71% for the group combining therapies compared to cirrhotic controls “Fig 3D”.

### 3.5 Functional recovery from liver damage by hADSCs transplantation, Ad-huPA transduction and the combined therapies

**Fig 5:**
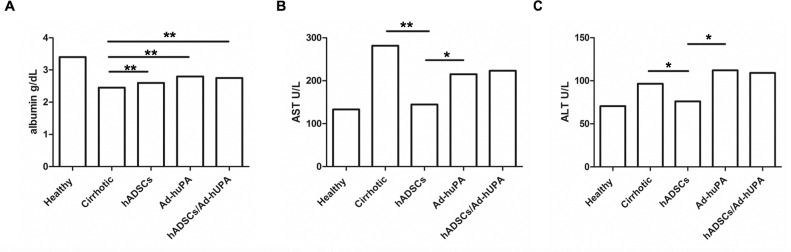
Serum levels of hepatic enzymes. (A) Liver synthesized albumin augmented in groups that receive cell or/and gene therapy compared to cirrhotic group. **(B,C)**Transaminases (ALT and AST) showed a diminution only in animals treated with hADSCs. Data represent the median for each group (*P<0.05, **P<0.01, ***P<0.001).

We evaluated the effect of therapies on the extent of liver function. Serum level of albumin, which is an indicator of liver function, increased in treated groups to 2.7±0.26 hADSCs, 2.8±0.31 Ad-huPA and 2.7± 0.22 g/dl hADSCs/Ad-huPA (P>0.01; “Fig 5A”) compared to cirrhotic controls (2.4±0.15g/dl). Serum transaminase levels, which increase when the liver is damaged (AST 291.2±54.2 U/L, ALT 94.8±15.4 U/L) were attenuated by hADSCs treatment (AST 173.5±50.6 U/L, ALT 76.5±12.8 U/L; P>0.05). Whereas AST showed a slight diminution in Ad-huPA (AST 239.5±53.0 U/L) and hADSCs/Ad-huPA groups (AST 235.8±92.5 U/L) “Fig 5B”.

### 3.6 Expression of profibrogenic molecules decreased by cell and gene therapy and their combination

We tried to elucidate the mechanistic basis of the anti-fibrogenic effect of individual and combined therapies. Given that hADSCs were reported to downregulate TGF-β1 and Col-1, we assessed the expression of TGF-β1, Col-1, CTGF, and other molecules previously reported to be modified in Ad-huPA treated animals, such as MMP2, α-SMA and PAI-I.

Expressions of TGF-β1, Col-1, CTGF, MMP2, α-SMA and PAI-I were down-regulated by hADSCs, Ad-huPA and hADSCs/Ad-huPA treatments compared to cirrhotic controls. For Col-1, α-SMA and MMP2, no statistical difference between treatments was observed. “Fig 4”. Lowest mRNA levels of PAI-I were found in the hADSCs group, whereas CTGF decreased mainly in the Ad-huPA group. Decrease in hepatic mRNA levels of Col-1 after treatments correlates with the diminution observed in collagen-sirius red staining; this data together with the diminish in TGF-β1 mRNA suggested ECM remodeling following hepatic hADSCs homing and Ad-uPA transduction.

## 4. Discussion

In this study we show that systemic application of hADSCs, Ad-huPA, or hADSCs/Ad-huPA results in the amelioration of liver fibrosis, reducing the amount of fibrotic tissue. Furthermore, expression of profibrogenic molecules decrease, whereas hepatic cell function improves.

Fibrosis regression achieved by gene therapy is comparable to that obtained in previous publications using Ad-huPA[12]. As it is known, uPA protein cleaves pro-HGF to its active form inducing ECM degradation releasing growth factors and MMPs retained in ECM [18,19]. In this study we evaluated necroinflammation index and α-SMA staining; both parameters are also reduced in this group of animals, indicating extensive improvement in hepatic tissue due to treatment.

Regarding the application of hADSCs into fibrotic rats, our data are similar to those studies performing systemic hADSCs transplantation (in our case, iliac vein) resulting in a substantial fibrotic tissue reduction. In addition, hADSCs cell therapy in our hands, improves serum hepatic enzymes, reduces liver α-SMA staining area and mRNA, and decreases Col-α1, TGF-β1, CTGF, PAI-I and MMP2 mRNA levels. These results indicate a clear beneficial effect of hADSC transplantation in liver damage. We believe that hADSCs have clear advantages over hBMSCs as a cell transplantation source. However, their transplantation in advanced liver fibrosis models have shown some discrepancies. Kamada et al. reported that spleen administration of 1x10^5^ GFP-positive mADSCs in CCl_4_-injured mice increased the area of liver fibrosis and Col-Iα1 mRNA levels, but reduced ALT and increased Albumin 4 weeks after transplantation. When the same cells are pre-treated with bFGF prior to transplantation, anti-fibrogenic effects were shown [2]. In contrast, other authors like Harn et al. reported a 1.9 fold fibrotic tissue reduction and hepatic function improvement after 1x10^6^ hADSCs administration, 14 days post-treatment in TAA cirrhotic rats [3]. Lee et al. transplanted 1X10^6^ hADSCs into DMN-cirrhotic rats via tail vein; 7 day after treatment fibrotic area reduced 3.7 fold [4]. The administration route also seems to influence the antifibrotic effect. Wang et al. proved portal vein or tail vein administration of 2x10^6^ rADSCs in CCl_4_-cirrhotic animals. Six weeks after treatment only the portal vein group reduced fibrotic tissue 2.3 fold [5]. As can be observed, cell origin, administrations routes, hepatotoxic and even animal strain employed varies in reported studies, but most of them agree in a helpful effect for liver fibrosis cell-based therapies. Mechanisms proposed for the ADSC antifibrotic effect include the secretion of IL-10 and HGF, which have a paracrine influence on activated hepatic stellate cells (HSC) [10]. This assumption correlates with our results for hepatic α-SMA staining and mRNA levels that diminish, probably due to a decrease in HSC activation. To our knowledge, few studies have reported cell tracking in cirrhotic animals. Liver, spleen and lung are the organs that usually retain transplanted cells [20]. Outstandingly, we were able to detect DAPI-labeled hADSCs in fibrotic liver. We consider that biodistribution observed in this study supports a safe and efficient use of hADSCs for liver diseases, when administered in a similar vein, since cells “homing”, principally in liver tissue. hADSCs are known to be chemoattracted to damaged tissue, due to the expression of SDF-1α, PDGF-AB, TGF-β1 and TNF-α; molecules broadly overexpressed in fibrotic liver [21]. However, the aim of this study was to search for an enhanced antifibrogenic effect when cell therapy and gene therapy were simultaneously administrated. Contrary to our expectations, the combination of therapies does not substantially increase beneficial properties of individual therapies; even when a slight increase in fibrosis reversion is demonstrated when compared with gene therapy alone (p<0.05). Since the expression of the transgene (huPA) is similar in Ad-uPA and hADSCs/Ad-uPA groups, we assume that cell therapy does not interfere with liver transduction of Ad-huPA. Conversely, it seems that Ad-huPA transduction interferes with hADSCs liver therapy since cell therapy alone, shows enhanced antifibrogenic outcome than the combination of cell and gene therapy. As previously reported, immune response is provoked after adenovirus systemic administration [22]. Besides, hADSCs are known to display anti-inflammatory and immunoregulatory properties [23,24]. Hence, ADSCs therapeutic effect could be attenuated trying to neutralize adenovector-induced transient inflammation and reducing fibrosis and necroinflammation due to CCl_4_ intoxication. Our results show that liver inflammation after hADSCs transplantation was also reduced, probably due to PGE2 and IL-10, cytokines also known to be secreted by ADSCs [10,24]. However, more experiments should be run to corroborate this hypothesis. When MSCs are transduced prior to transplantation with antifibrogenic transgenes, they have exerted an improved antifibrogenic effect compared to un-modified MSC [25,26,20], with no apparent interference due to transgene expression. Therefore, this option could be explored with Ad-uPA.

## 5. Conclusions

In conclusion, cell therapy using ADSCs gene therapy with Ad-huPA and their simultaneous administration have shown substantial antifibrogenic results in a rat cirrhosis model, but conditions (time of administration, route and dose) for optimizing the conjunction of therapies must be evaluated.

## Supporting Information

S1 File List of Commercial Taqman probe/primers for real time PCR. (DOCX)

S2 File Data regarding PCR, Masson, Sirius red and IHC Staining, and biochemical parameters. (XLS)
