# Supplementary material for: Simultaneous Administration of ADSCs-Based Therapy and Gene Therapy Using Ad-huPA Reduces Experimental Liver Fibrosis
Source: PLoS One. 2016 Dec 16;11(12):e0166849. doi: 10.1371/journal.pone.0166849 (PMC5161330; doi:10.1371/journal.pone.0166849)
Supplement: S1 File — (DOCX) [file pone.0166849.s001.docx]

Molecule Probe sequence Catalog number

GAPDH AAACCCATCACCATCTTCCAGGAGC Rn99999916_s1

COL1A2 GAGCTGCTGGCCCATCTGGTCCTAA Rn00670303_g1

TGFB1 ACCGCAACAACGCAATCTATGACAA Rn00572012_m1

CTGF CCCTGCCCTAGCTGCCTACCGACTG Rn01537278_g1

MMP2 acaggtcccttgctggtggccaca Rn02532334_S1

ACTA2 ACGTACAACTGGTATTGTGCTGGAC Rn01759928_G1

PAI1A TCTTCCTCCACAGCCATTCTAGTCT Rn01481341_m1

**Box 1.** List of commercial Taqman probe/primers for real time PCR (Life Technologies). GAPDH: glyceraldehyde-3-phosphate dehydrogenase; COL1A2: collagen, type I, alpha 2 chain; TGFB1: transforming growth factor-β1; CTGF: connective tissue growth factor; MMP2: matrix metallopeptidase 2; ACTA2: smooth muscle alpha-actin 1; PAI1A: plasminogen activator inhibitor type
